# Predictors of health-related quality of life in patients with colorectal cancer

**DOI:** 10.1186/1477-7525-6-66

**Published:** 2008-08-25

**Authors:** Kathleen J Yost, Elizabeth A Hahn, Alan M Zaslavsky, John Z Ayanian, Dee W West

**Affiliations:** 1 Center on Outcomes, Research and Education, Evanston Northwestern Healthcare, Evanston, IL 60201, USA; 2 Department of Preventive Medicine, Feinberg School of Medicine, Northwestern University, Chicago, IL 60611, USA; 3 Department of Health Care Policy, Harvard Medical School, Boston, MA 02115, USA; 4 Division of General Medicine, Brigham and Women's Hospital, Boston, MA 02115, USA; 5 Northern California Cancer Center, Fremont, CA 94538, USA

## Abstract

**Background:**

Most studies that have identified variables associated with the health-related quality of life (HRQL) of patients with colorectal cancer have been cross-sectional or included patients with other diagnoses. The objectives of this study were to identify predictors of HRQL in patients with colorectal cancer and interpret the clinical importance of the results.

**Methods:**

We conducted a population-based longitudinal study of patients identified through three regions of the California Cancer Registry. Surveys were completed by 568 patients approximately 9 and 19 months post-diagnosis. Three HRQL outcomes from the Functional Assessment of Cancer Therapy – Colorectal (FACT-C) were evaluated: social/family well-being (SWB), emotional well-being (EWB) and the Trial Outcome Index (TOI), which is a colorectal cancer-specific summary measure of physical function and well-being. Sociodemographic, cancer/health, and healthcare variables were assessed in multivariable regression models. We computed the difference in predicted HRQL scores corresponding to a large difference in a predictor variable, defined as a 1 standard deviation difference for interval variables or the difference relative to the reference category for nominal variables. The effect of an explanatory variable on HRQL was considered clinically meaningful if the predicted score difference was at least as large as the minimally important difference.

**Results:**

Common predictors of better TOI, SWB and EWB were better general health and factors related to better perceived quality of cancer care. Predictor variables in addition to general health and perceived quality of care were identified only for SWB. Being married/living as married was associated with better SWB, whereas being male or of Hispanic ethnicity was associated with worse SWB. Among the sociodemographic, cancer/health, and healthcare variables evaluated, only Hispanic ethnicity had a clinically meaningful effect on an HRQL outcome.

**Conclusion:**

Our findings, particularly the information on the clinical importance of predictor variables, can help clinicians identify patients who may be at risk for poor future HRQL. Potentially modifiable factors were related to perceived quality of cancer care; thus, future research should evaluate whether improving these factors improves HRQL.

## Background

Most patients with colorectal cancer survive at least five years after diagnosis [1], making health-related quality of life (HRQL) an important outcome for these patients. Patient-reported outcomes including HRQL have been used in conjunction with traditional clinical outcomes, such as treatment response rates and disease-free survival, to assess treatment efficacy in randomized clinical trials [2-4]. HRQL is also used in quality of care research [5] and is a predictor of survival of patients with colorectal cancer [6-8]. Understanding the characteristics or conditions that predict subsequent HRQL may help clinicians identify patients who are at risk for poor HRQL. Furthermore, if a characteristic or condition is modifiable, an intervention to alter it could lead to improved HRQL.

Most studies of HRQL of patients with colorectal cancer have been cross-sectional [9-11]. While cross-sectional analyses are valuable, longitudinal analyses of predictors of HRQL are needed to understand how HRQL changes over time [11]. Previous studies of HRQL that combined data from patients with colorectal cancer and patients with other diagnoses such as breast or lung cancer [12-14] may have failed to identify relationships involving disease- and treatment-related side-effects specific to patients with colorectal cancer. Furthermore, previous research tends to focus on the HRQL of cancer patients while in treatment [15] or the HRQL of cancer survivors several years post-diagnosis [16-18]. Less is known about HRQL during the period when patients have completed treatment and are transitioning to the survivorship phase. A final limitation of previous research on predictors of HRQL is that it has not considered whether the variables identified based on statistical criteria are also clinically meaningful. Therefore, our study had the following two objectives: (1) to identify variables that predict HRQL in a prospective, population-based study of patients with colorectal cancer, and (2) to interpret the regression results in the context of clinical importance.

## Methods

### Study population

Men and women aged 40 to 84 diagnosed with invasive (i.e., excluding *in situ*) colorectal cancer from April 1999 through June 2000 were identified from three of the ten regional registries that comprise the statewide population-based California Cancer Registry (CCR): Region 1 (San Jose/Monterey area), Region 3 (Sacramento area), and Region 8 (San Francisco/Oakland area) [19].

### Timing of assessments

This study employed rapid case ascertainment to identify eligible patients within 3 to 6 months of diagnosis. Respondents were first assessed in an initial survey an average of 9.2 months (range 4.6 – 23.0 months; SD 2.6 months) post-diagnosis when most patients would have recovered from their cancer surgery and completed their adjuvant treatment, if any. The primary purpose of the initial survey was to assess the quality of care for colorectal cancer by hospital and patient characteristics. The initial survey, as previously described [20], was completed by 1,079 English- or Spanish-speaking respondents (response rate 72.4%).

Patients were eligible for participation in the follow-up survey if they completed the initial survey and were English-speaking. A total of 830 English-speaking respondents in the initial survey were invited to participate in the follow-up survey an average of 19.1 months (range 13.0 – 31.8 months; SD 2.7 months) after diagnosis when most patients would be in a relatively stable disease state. The average time between the initial and follow-up surveys was 10.2 months (range 4.7 – 17.4 months; SD 1.8 months). Data for both the initial and follow-up surveys were collected predominantly via telephone by trained interviewers from the California Public Health Institute's Survey Research Group. Some patients were surveyed through a mailed, self-administered questionnaire, particularly patients who were hearing-impaired or not successfully contacted by telephone. Institutional review boards of the California Department of Health Services, Public Health Institute, Harvard Medical School, and the Northern California Cancer Center approved the study protocols for the initial and follow-up surveys. Participant consent was obtained prior to conducting the surveys.

### Surveys

HRQL was measured in the initial and follow-up surveys with the Functional Assessment of Cancer Therapy-Colorectal (FACT-C) [21]. The FACT-C is a valid and reliable measure of HRQL and includes five subscales: Physical Well-Being (PWB, 7-items), Functional Well-Being (FWB, 7-items), Social/family Well-Being (SWB, 7-items), Emotional Well-Being (EWB, 6-items), and the Colorectal Cancer Subscale (CCS, 7-items), which measures concerns specific to colorectal cancer patients such as appetite and bowel control [21]. The FACT-C was scored as described in the documentation for the instrument, with higher scores indicating better HRQL [22]. The Trial Outcome Index (TOI), derived as the sum of the PWB, FWB, and CCS subscales, is a summary measure of physical function and well-being in colorectal cancer patients that is useful as a patient-reported outcome in pharmacologic interventions [21]. The TOI and the remaining two subscales, SWB and EWB, were the HRQL outcomes evaluated in this study.

Patients' perceived quality of cancer care was measured using 31 items obtained from the Picker Institute of Boston, Massachusetts [20,23,24] that measure problems associated with six aspects of cancer care: psychosocial care, access to cancer care, treatment information, health information, confidence in providers, and coordination of care. Two items measuring patients' perceptions of how well healthcare providers controlled their pain/discomfort and nausea/vomiting were also included. Higher scores indicate greater perceived problems with care.

Limitations due to each of 14 comorbid conditions were measured in the initial survey and scored as 0 if the condition was not present, 1 if the condition was present but did not limit the patient, or 2 if the condition was present and limited the patient. Scores were summed to create a comorbidity index (range 0 to 28) [12,25]. General health was measured with a single item from the Medical Outcomes Study 36-item Short form (SF-36) health survey [26]. Bowel function and overall bowel problems were measured with items from the Prostate Cancer Outcomes Study [27]. Respondents who had a colostomy at the time of the interviews were not asked these bowel-related items, including 131 (12.1%) of 1,079 patients in the initial survey and 54 (9.5%) of 568 patients in the follow-up survey.

Sociodemographic information collected via survey included race/ethnicity, education, household income, financial difficulty due to cancer, occupational status, marital status, and number of persons living in the household. Gender, age at diagnosis and stage at diagnosis were obtained from the CCR. Neighborhood socioeconomic status (SES) was calculated based on 2000 U.S. Census data using methods described in Yost et al. [28].

### Data analyses

#### Non-response bias

To assess potential non-response bias, characteristics of eligible patients who did not participate in the follow-up survey were compared to those of patients who did using standard statistical tests for continuous and categorical data. All candidate predictor variables for the longitudinal analyses were measured either at the time of diagnosis or during the initial survey.

#### Regression models

Because previous studies of predictors of HRQL are inconclusive [11-13,17,29-31], we adopted an exploratory approach to identify variables predictive of HRQL at the follow-up survey. The range of time since diagnosis for the initial survey (4.6 – 23.0 months) overlapped with that for the follow-up survey (13.0 – 31.8 months). Therefore, we created non-overlapping time periods for each survey centered around the means (i.e., 9 months for initial survey and 19 months for the follow-up survey). The time period for the initial survey was restricted from 4 to less than 14 months post-diagnosis and the time period for the follow-up survey was restricted from 14 to less than 24 months post-diagnosis. The regression analyses were restricted to participants who completed their initial and follow-up surveys within these ranges. This resulted in the exclusion of 43 participants who were missing data for time since diagnosis for either the initial or follow-up survey and 29 participants who were outside of the restricted time frames. Following these exclusions, the mean times since diagnosis for the 496 respondents were 8.6 months (range 4.6–13.9, SD 1.7) for the initial survey and 18.8 months (range 14.0–24.0, SD 2.1) for the follow-up survey.

Separate linear regression analyses [32] were conducted for each of the three HRQL outcomes measured at the follow-up survey using the following candidate predictor variables. **Initial HRQL**: TOI, SWB or EWB measured at the initial survey. **Sociodemographic**: age at diagnosis, gender, race/ethnicity, marital status (married/living as married vs. not married), education (high school or less vs. technical school or some college, college or higher), occupational status (working vs. not working), number in household, neighborhood SES (standardized principal component score [28]), financial problems due to cancer, and household income (missing, <$25,000, $25,000–$50,000 vs. $50,000+). Household income was not reported by 58 (10.2%) respondents. Rather than exclude these respondents from the analyses, "missing" income was treated as a separate income category. **Cancer/health**: stage at diagnosis (Stage I/II/III vs. Stage IV), general health, colostomy (yes/no), history of radiation therapy (yes/no), history of chemotherapy (yes/no), currently receiving chemotherapy at the time of the initial survey (yes/no), comorbidity index, bowel function, overall bowel problems, family history of colorectal cancer (yes/no), time since diagnosis, and site (colon vs. rectum). **Healthcare**: type of health insurance [Medicare, other/none (e.g., Medicaid, other government-provided, uninsured) vs. commercial (e.g., HMO, PPO, private)], six domains of perceived quality of care, control of pain and discomfort (definitely vs. somewhat/not at all) and control of nausea and vomiting (definitely vs. somewhat/not at all).

Variables associated with HRQL were selected in two stages. First, follow-up HRQL was regressed on initial HRQL plus one other candidate variable. Candidate variables that had a significant relationship with follow-up HRQL by a liberal (*p *< 0.25) criterion were identified [33]. These variables were then combined into a multivariable model. Backward elimination with a criterion of *p *< 0.05 for retention was used to select a final model. We also conducted forward and stepwise regression to determine whether the same model was identified. Multicollinearity in the final models was assessed with the variance inflation factor (VIF). To facilitate interpretation of our regression results, we report squared semi-partial correlations (*sr*^2^) in addition to *p*-values. Because the *sr*^2 ^expresses the unique variance in the dependent variable explained by a predictor variable, it is a useful measure of the importance of a predictor [34].

#### Meaningful effects

We assessed the clinical meaningfulness of each predictor variable [35,36]. For interval variables (e.g., age), we computed the difference in predicted HRQL scores corresponding to a large difference in a predictor variable, defined as a 1 SD difference [37], where the SD was based on the data for the 496 respondents evaluated in the regression analyses. For nominal variables (e.g., race/ethnicity), we computed the difference in the predicted HRQL score relative to the reference category. The effect of an explanatory variable on follow-up HRQL was considered meaningful if the corresponding predicted score difference was at least as large as the minimally important difference (MID), which we defined as the smallest difference in HRQL scores that patients perceive as important, and thus might lead a clinician to consider changing the patient's management [38]. MIDs have been determined for the TOI (4–6 points) [39] and the SWB and EWB subscales (2–3 points) [40]. We used the lower bounds of these ranges to identify clinically meaningful effects as an indication of the potential prognostic impact of predictor variables. We also computed the percent of patients whose HRQL improved or declined more than the lower bound of the MID range.

## Results

### Sample characteristics

Of the 830 English-speaking patients invited to participate in the follow-up study, 26 were ineligible because they had died. Follow-up surveys were completed by 568 (70.6%) of the 804 eligible patients. Of the 236 patients who did not participate in the follow-up study, 28 were either hearing or mentally impaired or too ill to participate, 79 refused, and 129 were not successfully contacted.

Table 1 summarizes the characteristic at the time of diagnosis or at the time of the initial survey for the 496 participants of the follow-up survey with non-overlapping survey periods who were evaluated in this study. Characteristics for all 568 respondents and the 236 non-respondents of the follow-up survey are also described in Table 1. Non-respondents were significantly more likely to be non-white, unmarried, with low income, more financial problems due to cancer, longer time since diagnosis, have more perceived problems with access to care, confidence in providers, and coordination of care, and more perceived problems with control of pain/discomfort. They were significantly less likely to be on chemotherapy at the time of the initial survey. HRQL scores measured in the initial survey were lower for non-respondents than for respondents of the follow-up survey. Cronbach's alpha, a measure of reliability, was acceptable (≥0.70) in both the initial and follow-up surveys, respectively, for the TOI scale (0.90, 0.90) and the SWB (0.74, 0.77) and EWB (0.74, 0.76) subscales. The 95% confidence intervals around the initial scores for SWB (22.9, 23.7) and EWB (20.1, 20.7) for the 568 respondents did not contain the general U.S. population norms of 19.1 and 19.9 for the SWB and EWB, respectively [41], nor did they contain the norms for colon cancer patients of 22.0 and 19.8, respectively [22]. This indicates that the respondents had significantly higher scores for these domains than the norms. There are no general or cancer-specific norms for the TOI.

**Table 1:** Characteristics of follow-up survey respondents and non-respondents at the time of diagnosis or the initial survey

**Variable Type**	**Variable^a^**	**Evaluated** **Respondents** ** (*n *= 496)^b,c^**	**Assessment of Response Bias^b^**		
			
			**Follow-up** **Survey** **Respondents** ** (*n *= 568)**	**Follow-up** **Survey** **Non-Respondents** ** (*n *= 236)**	***p*-value^d^**
Sociodemographic	Age at diagnosis (years) [mean (SD)]	66.8 (10.4)	66.7 (10.5)	65.0 (12.1)	0.07
	Male	48.0	48.6	50.0	0.72
	Race/ethnicity				
	Non-Hispanic White	80.0	79.8	67.8	<0.001
	Non-Hispanic Black	5.7	5.6	14.0	
	Hispanic	7.5	7.0	9.3	
	Asian/Other	6.9	7.6	8.9	
	Married/Living as married	65.9	65.7	56.6	0.02
	Education				
	High school or less	38.1	38.2	44.0	0.30
	Post high school training/some college	30.7	31.1	27.8	
	College degree or higher	31.1	30.7	28.2	
	Working	28.6	28.8	29.7	0.80
	Household Income				
	Missing	8.9	10.2	11.9	<0.001
	Less than $25,000	22.4	23.1	36.0	
	$25,000 to $50,000	31.1	30.3	20.8	
	Over $50,000	37.7	36.4	31.4	
	Financial problems due to cancer				
	Not at all	76.6	77.2	66.4	0.01
	A little	10.9	10.2	16.4	
	Somewhat	7.3	7.1	11.2	
	A lot	5.0	5.5	6.0	

Cancer/Health-related	Late Stage (Stage IV)	8.3	7.8	10.2	0.26
	General Health				
	Poor	5.0	5.3	5.1	0.21
	Fair	23.2	22.4	23.3	
	Good	38.7	38.2	41.1	
	Very Good	21.2	22.7	24.6	
	Excellent	11.9	11.4	5.9	
	Colostomy	14.5	14.5	15.7	0.67
	History of radiation therapy	15.3	17.4	19.4	0.51
	History of chemotherapy	51.6	49.2	42.0	0.07
	Receiving chemotherapy at time of initial survey	30.9	30.1	19.5	0.002
	Comorbidity Index [median (IQR)]	2 (1–4)	2 (1–4)	3 (1–4)	0.08
	Bowel Function [mean (SD)]	8.6 (2.3)	8.6 (2.3)	8.8 (2.4)	0.42
	Time since diagnosis [mean (SD)]	8.6 (1.7)	9.2 (2.6)	9.8 (3.1)	0.02

Healthcare	Type of Insurance				
	Medicare	24.2	24.3	28.8	0.40
	Commercial Insurance	68.2	68.0	64.4	
	Other/none	7.7	7.8	6.8	
	Picker problem scores [mean, (SD)]				
	Psychosocial Care	30.6 (28.9)	30.7 (28.5)	32.4 (28.1)	0.45
	Access to Care	10.8 (22.5)	10.7 (22.4)	16.7 (26.9)	0.003
	Treatment Information	30.4 (30.7)	30.5 (30.9)	33.9 (33.6)	0.17
	Health Information	46.1 (33.6)	46.6 (33.6)	49.6 (35.7)	0.25
	Confidence in Providers	11.7 (20.6)	11.8 (21.4)	15.5 (26.7)	0.05
	Coordination of Care	19.4 (23.5)	19.0 (23.5)	24.4 (27.4)	0.008
	Control of Nausea/Vomiting (% somewhat/not at all)	11.3	10.9	14.0	0.22
	Control of Pain (% somewhat/not at all)	10.3	10.4	18.2	0.002

HRQL	Initial HRQL scores [mean, (SD)]				
	TOI	66.6 (12.7)	66.9 (12.7)	64.6 (13.7)	0.02
	SWB	23.2 (4.4)	23.2 (4.6)	22.0 (5.1)	0.002
	EWB	20.4 (3.7)	20.4 (3.7)	19.6 (4.5)	0.02

HRQL, Health-related Quality of Life; TOI, Trial Outcome Index-Colorectal; SWB, Social/family well-being; EWB, Emotional well-being; SD, standard deviation; IQR, interquartile range.

^a^Data for all variables were collected via self report in the initial survey except gender, stage at diagnosis and age, which were reported to the California Cancer Registry at the time of diagnosis.

^b^Numbers represent percentages unless otherwise specified.

^c^Participants with non-overlapping survey periods who were evaluated in the regression analyses.

^d^*p*-value for response bias at follow-up. Compares follow-up respondents (*n *= 568) and non-respondents (*n *= 236).

### Clinically meaningful change in HRQL

The percent of patients with a clinically meaningful decline in EWB scores from the initial to follow-up survey was exactly the same as the percent with a clinically meaningful improvement (Table 2). Slightly more patients had a meaningful improvement than decline in TOI scores, but fewer patients had a meaningful improvement than decline in SWB scores. EWB scores were the most stable, with 47.6% of patients experiencing a change less than the MID. The magnitude of the average decline in scores was slightly larger than that of the average improvement for all three HRQL outcomes.

**Table 2:** Clinically meaningful change in HRQL* from the initial to follow-up surveys

**Change in HRQL**	**TOI**		**SWB**		**EWB**	
	**n (%)**	**mean (SD)**	**n (%)**	**mean (SD)**	**n (%)**	**mean (SD)**
Meaningful Decline	161 (32.5)	-11.0 (6.2)	167 (33.7)	-5.2 (3.2)	130 (26.2)	-4.5 (2.7)
About the same	163 (32.9)	.07 (2.0)	193 (38.9)	-.15 (.85)	236 (47.6)	.05 (.70)
Meaningful Improvement	172 (34.7)	10.5 (7.3)	125 (25.2)	4.2 (2.4)	130 (26.2)	3.8 (2.2)

HRQL, Health-related Quality of Life; TOI, Trial Outcome Index-Colorectal; SWB, Social/family Well-being; EWB, Emotional Well-being

*Clinically meaningful change is at least 4 points for the TOI and at least 2 points for SWB and EWB.

### Prognostic impact on patient-reported HRQL

#### Predictors of follow-up TOI scores

In addition to initial TOI scores, two variables were retained as predictors of follow-up TOI following backward elimination (Table 3), accounting for 43.9% of the variance. Forward and stepwise selection identified the same model. No sociodemographic variables were retained. One cancer/health-related variable, general health, was retained. The Treatment Information problem score from the Picker Institute measure was the only healthcare variable retained. Only initial TOI was a clinically meaningful predictor of follow-up TOI, with a 1 SD increase (12.7 points) in initial TOI scores corresponding to a 6.4 point increase in follow-up TOI scores. Initial TOI accounted for the largest proportion of variance, as indicated by the *sr*^2^. The *p*-value, *sr*^2 ^and effect on follow-up TOI indicated that general health had a greater prognostic impact than Treatment Information. All VIFs were less than 2.0 indicating there was no multicolinearity among the predictors.

**Table 3:** Predictors of follow-up TOI

**Variable Type**	**Variable**	**B (SE)**	***p*-value**	***sr***^2^	**Effect on** **follow-up** HRQL*
HRQL	Initial TOI	.50 (.04)	<.0001	.179	**6.39**
Cancer/Health-related	General health	1.88 (.48)	<.0001	.018	1.98
Healthcare	Treatment information problem score	-.04 (.01)	.005	.009	-1.18

Adjusted R^2^: 43.9					

HRQL, Health-related Quality of Life; TOI, Trial Outcome Index-Colorectal; **B**, regression coefficient; **SE**, standard error; ***sr***^2^, squared semi-partial correlation

*Difference in predicted follow-up TOI score for a 1 SD difference in interval variables or the difference relative to the reference category for nominal variables. Effects with a magnitude of at least 4 points are considered meaningful and are indicated in bold font.

#### Predictors of follow-up SWB scores

Backward selection identified a model with six statistically significant predictors of follow-up SWB explaining a total of 39.3% of the variance (Table 4), including initial SWB, three sociodemographic indicators (gender, race/ethnicity, marital status), one cancer/health-related indicator (general health), and one healthcare measure (problems with control of pain/discomfort). Forward and stepwise selection yielded a slightly different model that did not include gender and marital status. We report the model identified using backward selection as it had a larger adjusted R^2 ^(39.3 vs. 38.1). Both initial SWB and Hispanic ethnicity were clinically meaningful predictors of follow-up SWB. Hispanic ethnicity also had a larger *sr*^2 ^relative to the other sociodemographic, cancer/health and healthcare variables.

**Table 4:** Predictors of follow-up SWB

**Variable Type**	**Variable**	**B (SE)**	***p*-value**	***sr***^2^	**Effect on** **follow-up** HRQL*
HRQL	Initial SWB	.59 (.04)	<.0001	.222	**2.59**
Sociodemographic	Male	-1.08 (.39)	.006	.010	-1.08
	Race/ethnicity (ref = White)				
	Black	-.90 (.78)	.25	.002	-.90
	Hispanic	-2.49 (.69)	.0003	.016	**-2.49**
	Asian/other	-1.15 (.73)	.11	.003	-1.15
	Married/living as married	1.10 (.41)	.008	.009	1.10
Cancer/Health-related	General health	.48 (.18)	.009	.009	.51
Healthcare	Problems with control of pain/discomfort	-1.43 (.61)	.02	.007	-1.43

Adjusted R^2^: 39.3					

HRQL, Health-related Quality of Life; SWB, Social/family Well-being; **B**, regression coefficient; **SE**, standard error; ***sr***^2^, squared semi-partial correlation

*Difference in predicted follow-up SWB score for a 1 SD difference in interval variables or the difference relative to the reference category for nominal variables. Effects with a magnitude of at least 2 points are considered meaningful and are indicated in bold font.

#### Predictors of follow-up EWB scores

Predictors of EWB at the follow-up survey included initial EWB, general health and control of nausea/vomiting, explaining 36.5% of the variance (Table 5). Backward, forward and stepwise selection all identified the same model. After initial EWB, general health had the largest *sr*^2^, while problems with control of nausea/vomiting had the largest prognostic impact as indicated by the size of the effect on follow-up EWB. Initial EWB was the only meaningful predictor of follow-up EWB.

**Table 5:** Predictors of follow-up EWB

**Variable Type**	**Variable**	**B (SE)**	***p*-value**	***sr***^2^	**Effect on** **follow-up** HRQL*
HRQL	Initial EWB	.55 (.04)	<.0001	.210	**2.05**
Cancer/Health-related	General health	.54 (.15)	.0004	.017	.56
Healthcare	Problems with control of nausea/vomiting	-1.25 (.46)	.007	.009	-1.25

Adjusted R^2^: 36.5					

HRQL, Health-related Quality of Life; EWB, Emotional Well-being; **B**, regression coefficient; **SE**, standard error; ***sr***^2^, squared semi-partial correlation

*Difference in predicted follow-up EWB score for a 1 SD difference in interval variables or the difference relative to the reference category for nominal variables. Effects with a magnitude of at least 2 points are considered meaningful and are indicated in bold font.

## Discussion

Exploratory longitudinal analyses were conducted to evaluate the relationship between three HRQL outcomes and sociodemographic, cancer/health, and healthcare variables in a population-based sample of patients with colorectal cancer. General health was the only variable common to all three outcomes, although each model also contained a quality of care variable: Perceived problems with Treatment Information was a predictor of follow-up TOI, perceived problems with control of pain/discomfort predicted follow-up SWB and perceived problems with control of nausea/vomiting was a predictor of follow-up EWB.

Rather than relying solely on statistical measures such as *p*-values and *sr*^2 ^to interpret the results of the regression analyses, we also used clinical meaningfulness of the effect of a predictor variable on the HRQL outcome. For follow-up TOI, both initial TOI and general health were highly statistically significant predictors (*p *< 0.001). The *sr*^2 ^for general health was smaller than that for initial TOI, but even with this information, it may still not be intuitive to some clinicians or researchers whether to consider general health as an important predictor. By considering the clinical importance of these variables, we showed that even a large (1 SD) difference in the general health score would not have a clinically meaningful effect on follow-up TOI scores. This information may help clinicians and researchers understand the results regardless of their familiarity with regression modeling or the FACT-C instrument.

Perceived problems with Treatment Information was also retained in the model for follow-up TOI. A measure of perceived quality of treatment information is not commonly included in studies aimed at identifying predictors of HRQL. That this variable was identified in our study as a significant predictor of HRQL over other commonly evaluated variables such as gender, age, and comorbidities [12,17,30] warrants the addition of perceived quality of treatment information to the list of candidate variables in future research of HRQL predictors.

Relative to non-Hispanic White participants, Hispanic participants had significantly lower follow-up SWB. Wan et al. [14] also observed significantly lower SWB scores among Hispanic cancer patients relative to White patients. Both initial SWB and Hispanic ethnicity were highly significantly related to follow-up SWB (*p *< 0.001). The *sr*^2 ^for Hispanic ethnicity was much smaller than that for initial SWB suggesting that Hispanic ethnicity was the less influential predictor of the two. However, by considering clinical importance, we obtained a slightly different interpretation; that the two variables had comparable clinically meaningful effects on follow-up SWB.

The relationship between gender and the social domain of HRQL may depend on how the domain is measured. For example, as measured in the present study with the FACT-C, men had worse initial SWB (data not shown) and follow-up SWB than women. Normative data for the SWB in both the general U.S. population and cancer patients also show lower average scores for men than women [41]. However, several studies have evaluated the relationship between gender and social functioning as measured by the EORTC QLQ-C30 in cancer patients and reported no differences [31,42,43].

Although the association between being married/living as married and having better physical and psychological well-being is well established, the link between marital status and SWB is less clear [44]. We found that married/living as married persons had better SWB as measured by the FACT-C, which is predominantly a measure of social support and contains items such as "I get emotional support from my family" and "I feel close to my partner (or the person who is my main support)." It is reasonable to expect married/living as married persons to answer these questions more favorably.

Initial EWB was the only meaningful predictor of follow-up EWB. After initial EWB, general health was the strongest predictor based on the *p*-value and *sr*^2^, but problems with control of nausea and vomiting was the strongest predictor based on the effect on follow-up EWB score. Thus, by considering clinical importance, we obtained a slightly different interpretation of the importance of these two predictors.

The measures of perceived quality of care (problems with Treatment Information, control of pain/discomfort, control of nausea/vomiting) are the only predictors of HRQL in our study that are potentially modifiable. These variables are not typically included as predictors of HRQL in multivariable regression analyses, yet they were more clinically meaningful predictors of the HRQL outcomes than some more commonly evaluated predictors, including gender, marital status and general health. Additional research is needed to better understand the association between factors related to perceived quality of care at the time of cancer treatment and HRQL at some follow-up assessment. Furthermore, as these are potentially modifiable variables, intervention studies could explore methods for improving certain aspects of quality of care to determine whether those changes lead to improved HRQL.

While only a few of the statistically significant predictors individually met our criteria for clinical importance, in combination they may identify patients at high risk for poor HRQL. For example, unmarried male patients with worse general health and more perceived problems with control of pain/discomfort may have meaningfully lower average follow-up SWB scores (i.e., at least 2 points lower) than married female patients with better general health and fewer perceived problems with control of pain/discomfort even after adjusting for initial SWB. Initial HRQL was consistently the strongest predictor of follow-up HRQL; therefore, clinicians could identify patients at risk for poor future HRQL by routinely assessing HRQL in clinical practice [45].

A strength of our study is that the respondents were identified through a population-based cancer registry and were therefore representative of English-speaking colorectal cancer patients in California. A potential limitation of our study is possible non-response bias in the follow-up sample. The follow-up respondents and non-respondents differed significantly on a number of variables that may be related to follow-up HRQL, which limits the generalizability of our results. In particular, non-respondents had significantly lower initial HRQL than respondents and therefore likely had lower follow-up HRQL. Nonresponse might also have affected the selection and estimated prognostic impact of predictors of HRQL. Another potential limitation is that there may be other variables predictive of follow-up HRQL that were not considered in this analysis. Variables such as spiritual well-being [14,46], optimism [47], and sexual dysfunction [10] have been shown to be related to HRQL. However, these topics were not measured in the initial survey. The follow-up survey did not measure whether the respondents had experienced a recurrence or whether they were undergoing any adjuvant treatment for either their primary or a recurrent cancer, which could have affected their follow-up HRQL [29,48,49]. Because of inconclusive results in the literature regarding variables that predict HRQL in colorectal patients, we adopted an exploratory approach and evaluated a large number of variables, including variables rarely, if ever, evaluated previously in this population as potential predictors, such as perceived quality of cancer care. Thus, another potential limitation of our study is an inflated type I error. The predictors of HRQL in patients with colorectal cancer may differ from those in patients with other types of cancer. Furthermore, the predictors of HRQL may vary based on how HRQL is measured.

## Conclusion

We identified sociodemographic, clinical, and healthcare variables that predict HRQL as measured by the FACT-C. The most consistent finding was that patients with poor general health and problems with certain domains of perceived quality of cancer care may be at risk for poor HRQL. Other characteristics that might identify at-risk patients were specific to each HRQL outcome and included being male, unmarried or Hispanic. Computing the clinical importance of the effect on the HRQL outcome helped to interpret the impact of specific statistically significant predictors. As the only potentially modifiable variables identified in our study were related to perceived quality of cancer care, future research should evaluate whether interventions aimed at improving these variables enhances subsequent HRQL.

## List of abbreviations

HRQL: Health-related quality of life; FACT-C: Functional Assessment of Cancer Therapy-Colorectal; PWB: Physical well-being; EWB: Emotional well-being; SWB: Social/family well-being, FWB: Functional well-being; TOI: Trial outcome index; CCR: California Cancer Registry; SD: Standard deviation; HMO: Health maintenance organization; PPO: Preferred provider organization; VIF: Variance inflation factor.

## Competing interests

The authors declare that they have no competing interests.

## Authors' contributions

KJY, AMZ, JZA, and DWW made substantial contributions to the study concept and design, EAH and AMZ provided guidance on the statistical analysis, KJY conducted the statistical analysis, interpreted the results and drafted the manuscript, EAH, AMZ, JZA, and DWW participated in critical revisions of the manuscript for important intellectual content. All authors read and approved the final manuscript.
